# Chilaiditi Syndrome: A Rare Case of Chest Pain due to Colonic Interposition

**DOI:** 10.7759/cureus.9288

**Published:** 2020-07-20

**Authors:** Farhan Ali, Sowmya Srinivas, Hafiz Muzaffar Akbar Khan, Dayakar Reddy

**Affiliations:** 1 Internal Medicine, Arnot Ogden Medical Center, Elmira, USA; 2 Gastroenterology and Hepatology, Guthrie Robert Packer Hospital, Sayre, USA

**Keywords:** chilaiditi syndrome

## Abstract

We present an unusual case of Chilaiditi syndrome that manifests under the guise of multiple systemic signs and symptoms. An 81-year-old female patient with a history of coronary artery disease and hypothyroidism presented to emergency department (ED) with chest heaviness associated with nausea, shortness of breath, diffuse abdominal pain and constipation. Her symptoms were similar to the previous episode of ST-segment elevation myocardial infarction. The clinical team ruled out acute coronary syndrome based on electrocardiogram (EKG) and troponin levels. On further testing, CT of the abdomen revealed the interposition of colon on the dome of diaphragm consistent with Chilaiditi sign. The patient was diagnosed with Chilaiditi syndrome based on the characteristic radiological finding and the symptomatic presentation. Conservative management with bowel rest and laxative bowel regimen resolved her symptoms without further complications. A high index of suspicion is required for the early diagnosis and can prevent further complications and mitigate the need for laparoscopic intervention.

## Introduction

Chilaiditi syndrome is a rare condition characterized by a segmental interposition of the colon between the liver and the diaphragm [1]. Its incidence worldwide is estimated to be 0.025%-0.28% with 4:1 male predominance [2-5]. It is also noted to be more common in the elderly population [6]. It was first discovered by Cantini in 1865, and later described by Demetrius Chilaiditi, a Viennese radiologist in 1910 [2,4,5,7]. The exact etiology of this syndrome is unknown. We report a patient with Chilaiditi syndrome who presented to emergency department (ED) with symptoms of chest heaviness and abdominal pain and was found to have Chilaiditi syndrome diagnosed radiologically by CT.

## Case presentation

This clinical report describes the case of an 81-year-old female patient who presented to the ED with a past medical history significant for coronary artery disease status post previous myocardial infarction and hypothyroidism. She experienced chest heaviness for one hour that awoke her from sleep. Associated symptoms included nausea, shortness of breath, diffuse abdominal pain and constipation. She denied fever, chills, recent travel or sick contacts. On arrival to ED, she had a temperature of 98.40˚F, a blood pressure of 168/83 mm Hg, respiratory rate of 12/minute, heart rate of 108 bpm and oxygen saturation of 94% on 2 L oxygen. Upon examination, the patient was found to have abdominal distension and diffuse abdominal pain most prominent in the bilateral lower quadrants. No guarding or rigidity was noted. Her physical examination was otherwise unremarkable.

Electrocardiogram (EKG) performed was negative for ischemic changes or dysrhythmia. Chest x-ray did not show any evidence for acute cardiopulmonary disease. Serial drawn troponin enzyme levels were negative. The laboratory investigation included electrolytes, lipase, aspartate transaminase (AST), alanine transaminase (ALT), alkaline phosphatase, total bilirubin and thyroid-stimulating hormone (TSH), all of which were within normal limits.

**Figure 1:**
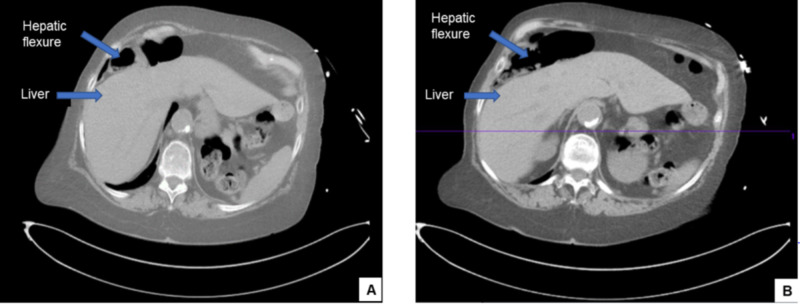
(A, B) CT scans of the abdomen reveals the interposition of hepatic flexure anteriorly in front of the liver up the dome of the diaphragm (Chilaiditi sign).

CT of the abdomen showed that the hepatic flexure was interposed anteriorly in front of the liver up to the dome of the diaphragm (Figure 1) consistent with ‘Chilaiditi sign’, a rare anatomical variant. Since the patient presented with symptoms, a diagnosis of Chilaiditi syndrome was made.

The patient was managed conservatively with laxative bowel regimen and bowel rest through her hospital course. Her symptoms of abdominal pain and chest discomfort resolved soon after her bowel movement, without recurrence for the duration of her hospital stay. She was subsequently discharged two days later without further complications

## Discussion

Chilaiditi syndrome manifests under the guise of multiple systemic signs and symptoms, including but not limited to abdominal pain, nausea, angina like chest pain, shortness of breath and constipation. Isolated reports have also chronicled clinical presentations similar to acute coronary syndrome, and upper respiratory illness [8]. In addition to a broad range of symptoms, Chilaiditi syndrome may mimic pneumoperitoneum on plain radiographs. Identification of rugal folds within the gas may help distinguish it from pneumoperitoneum [5,9]. Changing the patient’s position does not alter location of gas echo on film, in contrast to pneumoperitoneum [10].

Colonic interposition may also be episodic, which may add to the diagnostic challenge as patients may not have consistent radiographic evidence though their symptoms may be chronic in nature [11]. As a consequence, the diagnosis may be missed, and patients oftentimes become frustrated when confronted with such a broad constellation of symptoms. Not only can diagnosis be delayed, but so can the development of complications that would have otherwise been mitigated had the diagnosis been made earlier [7].

Although it proves to be difficult to diagnose, it is worth considering as part of a differential as a timely diagnosis may prevent the need for interventional procedures such as a laparotomy [12]. Risk factors include laxity of hepatic suspensory ligaments, liver atrophy from cirrhosis, diaphragmatic elevation from phrenic nerve injury, and elongation and hypermobility of the colon seen in patients suffering from constipation and obesity [2-5]. This syndrome is also associated with colonic, rectal and gastric malignancies.

Diagnosis is best made through abdominal CT as it is more sensitive than chest or abdominal radiography since it can be misread as pneumoperitoneum. Prevention is rooted in adopting healthy dietary regimen, in addition to close monitoring of individual metabolic profiles (e.g., blood sugar and lipid levels) [13].

Treatment is usually non-surgical, most commonly consisting of bowel rest, intravenous fluids, stool softeners and possible intervention by way of nasogastric decompression [4,10]. Colonoscopic evaluation should be avoided as the interposed bowel may be high risk for air entrapment and perforation [10]. Complications include intestinal obstruction and perforation. Invasive procedures include laparoscopic colonopexies, as well as colectomy, and hepatopexy [6]. Patients who have refractory symptoms, evidence of bowel ischemia or colonic volvulus require surgical intervention. Recently, Da Vinci robotic-assisted surgical intervention has been utilized to help reduce complications and shorten procedure time [9].

## Conclusions

Chilaiditi syndrome is a rare condition characterized by colonic interposition that is accompanied by a wide range of symptoms that include abdominal pain, chest pain, shortness of breath, constipation, nausea and vomiting. Due to its wide constellation of findings, diagnosis can be difficult. We propose that such symptoms should be considered in patients who have both acute and episodic occurrences of such symptoms. Timely diagnosis can prevent further complications and mitigate the need for laparoscopic intervention.
